# Survey of patient‐specific quality assurance practice for IMRT and VMAT

**DOI:** 10.1002/acm2.13294

**Published:** 2021-06-19

**Authors:** Gordon H. Chan, Lee C. L. Chin, Ady Abdellatif, Jean‐Pierre Bissonnette, Lesley Buckley, Daria Comsa, Dal Granville, Jenna King, Patrick L. Rapley, Aaron Vandermeer

**Affiliations:** ^1^ Department of Medical Physics Juravinski Cancer Centre Hamilton Ontario Canada; ^2^ Department of Medical Physics Odette Cancer Centre Toronto Ontario Canada; ^3^ Department of Medical Physics R.S. McLaughlin Durham Regional Cancer Centre Oshawa Ontario Canada; ^4^ Department of Medical Physics Princess Margaret Cancer Centre–UHN Toronto Ontario Canada; ^5^ Department of Medical Physics The Ottawa Hospital Ottawa Ontario Canada; ^6^ Radiation Physics Department Southlake Regional Cancer Centre Newmarket Ontario Canada; ^7^ Radiation Oncology Physics Simcoe Muskoka Regional Cancer Centre Barrie Ontario Canada; ^8^ Medical Physics Department Thunder Bay Regional Health Sciences Centre Thunder Bay Ontario Canada

**Keywords:** IMRT, PSQA, survey, VMAT

## Abstract

A first‐time survey across 15 cancer centers in Ontario, Canada, on the current practice of patient‐specific quality assurance (PSQA) for intensity modulated radiation therapy (IMRT) and volumetric modulated arc therapy (VMAT) delivery was conducted. The objectives were to assess the current state of PSQA practice, identify areas for potential improvement, and facilitate the continued improvement in standardization, consistency, efficacy, and efficiency of PSQA regionally. The survey asked 40 questions related to PSQA practice for IMRT/VMAT delivery. The questions addressed PSQA policy and procedure, delivery log evaluation, instrumentation, measurement setup and methodology, data analysis and interpretation, documentation, process, failure modes, and feedback. The focus of this survey was on PSQA activities related to routine IMRT/VMAT treatments on conventional linacs, including stereotactic body radiation therapy but excluding stereotactic radiosurgery. The participating centers were instructed to submit answers that reflected the collective view or opinion of their department and represented the most typical process practiced. The results of the survey provided a snapshot of the current state of PSQA practice in Ontario and demonstrated considerable variations in the practice. A large majority (80%) of centers performed PSQA measurements on all VMAT plans. Most employed pseudo‐3D array detectors with a true composite (TC) geometry. No standard approach was found for stopping or reducing frequency of measurements. The sole use of delivery log evaluation was not widely implemented, though most centers expressed interest in adopting this technology. All used the Gamma evaluation method for analyzing PSQA measurements; however, no universal approach was reported on how Gamma evaluation and pass determination criteria were determined. All or some PSQA results were reviewed regularly in two‐thirds of the centers. Planning related issues were considered the most frequent source for PSQA failures (40%), whereas the most frequent course of action for a failed PSQA was to review the result and decide whether to proceed to treatment.

## 1 INTRODUCTION

Patient‐specific quality assurance (PSQA) for static gantry intensity modulated radiation therapy (IMRT) and dynamic gantry volumetric modulated arc therapy (VMAT) delivery has been recognized as a key safety check to prevent radiotherapy delivery errors.^1^, ^2^, ^3^, ^4^, ^5^, ^6^ Only recently has the Medical Physics community published guidelines (e.g., Task Group 218^7^) to help streamline the PSQA process and the interpretation of the results.

The Ontario Health (Cancer Care Ontario) Physics Community of Practice (PCOP) is a community of “grass‐root” medical physicists with volunteer representatives from across Ontario Regional Cancer Centers with a shared purpose of identifying quality improvement initiatives pertaining to medical physics to improve the quality and safety of radiation treatment delivery. Prior to any new initiatives, the PCOP meets to identify high priority topics that are important to the Ontario medical physics community. It was recognized in early 2018 by the PCOP that the practice of PSQA can be disparate and the issue needed to be addressed. The primary motivation for this community‐driven PSQA initiative was to facilitate the continued improvement in standardization, consistency, efficacy, and efficiency of PSQA programs in Ontario.

The PCOP established a PSQA working group composed of nine medical physicists from centers of various sizes across the Canadian province of Ontario in January 2018. The working group was tasked to contrast the current state of PSQA practice in Ontario against existing guidance documents and relevant publications,^3^, ^4^, ^7^, ^8^, ^9^, ^10^ to identify areas for potential improvement, and to use gained knowledge to produce a provincial guidance document outlining best practice^11^ based on group consensus. To achieve these goals, the working group developed and distributed a survey covering a broad range of PSQA‐related topics to 15 cancer centers (14 regional and 1 affiliate centers) across Ontario. The key results of this survey are presented in this work.

It is important to know that in Ontario, at the time of survey, there is no legal requirement or financial incentive to carry out PSQA measurements or verification. Centers are free to follow any guidelines or use any PSQA methods they deem appropriate.

Several papers have reported the results of similar surveys on PSQA methods. In late 2017, a national survey on the practice of IMRT/VMAT in 403 centers in China collected data on medical physicists, equipment, IMRT/VMAT delivery and QA techniques, reimbursement, problems, and suggestions.^12^ The results showed that IMRT/VMAT QA was a significant burden, and variation existed in practice due to limited resources of physicists, QA devices, linacs and lack of national standards, technical guidelines, regulations, and training programs. The Imaging and Radiation Oncology Core (IROC)‐Houston QA center conducted a survey on IMRT/VMAT QA practice from 2011 to 2017 that included questions on treatment techniques, measurement devices and procedures, methods of calculating agreement, and strategies taken if PSQA fails.^13^ The vast majority of the responding 1455 site participants were from the United States and Canada. Although the practice used by most of those surveyed agreed with many of the TG‐218 recommendations, a notable percentage used delivery methods, evaluation criteria and strategies for dealing with PSQA failures that were not as rigorous as those recommended by TG‐218.^7^ In addition, a survey on planar IMRT QA analysis was published in 2007.^14^ However, it was limited to static gantry IMRT delivery and users of 2D diode array devices from a single vendor, and thus, their findings are less relevant to our study.

## 2 MATERIALS AND METHODS

The working group created a project charter for improving quality and safety in PSQA for IMRT delivery. One of the critical success factors was to create a comprehensive survey that captures the current state of PSQA practice. After conducting a review of the literature related to PSQA, individual group members contributed relevant survey questions, covering key topics of interest. The group as a whole then selected the questions that best aligned with the previously stated goals to be included in the survey. Technical term definitions were included in a glossary section to improve clarification.

The survey consisted of 40 multiple‐choice questions related to PSQA for IMRT and VMAT delivery. The answer choices were a list of probable answers that were determined collectively by the group members. For many questions, the choice, “Other,” was included to allow participants to write in their answers that were not in the list. For completeness, each question had a comment box allowing users to elaborate on their responses. The questions were grouped into six categories: policy and procedure; measurement vs delivery log; instrumentation; measurement setup and methodology; data analysis and interpretation; and documentation, process, and feedback. The primary focus of the survey was on routine photon radiation treatments using linacs, including linac‐based stereotactic body radiation therapy (SBRT) delivery. PSQA for stereotactic radiosurgery and/or specialized machines such as TomoTherapy^®^ or CyberKnife^®^ were considered out‐of‐scope. For this survey, PSQA was defined as either patient‐specific measurement or delivery log calculation, but not beam transfer check, predelivery dose verification, or secondary calculation of monitor units (MU). Delivery log refers to the record of beam parameters captured numerous times during a treatment delivery. These parameters can then be used to calculate the dose distribution delivered to a patient.

Survey questions, shown in the supporting information, were sent to participating centers using a web‐based commercial software (SurveyMonkey, San Mateo, CA). The working group estimated the time necessary to complete the survey would be 60 to 90 min. The survey was distributed to the heads of the medical physics departments at 15 cancer centers across Ontario in December of 2018, leveraging an existing and rather unique network of Ontario Health (Cancer Care Ontario) to encourage high participation and response rate. The size of the centers ranged from smaller centers with five or fewer physicists (five sites) to centers having more than 10 physicists (six sites). The number of physics assistants or associates ranged from one to six per center. The infrastructure at the sites included linacs from two major vendors: Elekta (Stockholm, Sweden) and Varian Medical Systems, Inc. (Palo Alto, CA) and four commercial treatment planning systems (TPS): Eclipse^TM^ (Varian), Monaco^®^ (Elekta), Pinnacle^3^ Treatment Planning (Philips, Amsterdam, Netherlands), and RayStation^®^ (RaySearch Laboratories, Stockholm, Sweden). As indicated in the survey instructions, only one response was allowed from each center. The working group indicated that the survey should be completed by the physicist(s) in charge of, or most familiar with, PSQA at each location. The participating centers were further instructed that their responses should reflect, as best as possible, the collective view or opinion of physicists at their site, and represent the most typical process practiced at the center.

All participating centers were asked to identify themselves at the beginning of the survey for the sole purpose of clarification about their responses or comments, if necessary, during follow up. Otherwise, individual center responses were de‐identified for all subsequent analysis and sharing of aggregated survey results. The final section of the survey document provided space for each respondent to offer general feedback to the working group about the survey.

The results of the survey were exported to a spreadsheet, and individual responses and comments were analyzed for accuracy, consistency, and clarity. After a preliminary review of all responses by the working group, some of the centers were contacted with the help of Ontario Health (Cancer Care Ontario) to protect anonymity with follow‐up questions in order to clarify selections or comments that raised additional questions, or appeared to conflict with prior answers from the same center. In nine questions, the working group changed or reclassified 15 initial responses based on the additional information and clarifying comments subsequently provided by the follow‐up, whereas responses were changed in three questions based strictly on written comments in the survey.

## 3 RESULTS AND DISCUSSION

All 15 centers (100% response rate) responded within 2 months of the survey distribution date. The data that support the findings of this study are available in the supporting information of this article. The summary of the responses in the six aforementioned categories is given below, with the question number referenced in brackets as appropriate. It is important to note that the participants were limited to the province of Ontario, and therefore, the sample size (15) is relatively small. The results of this survey reflect only the PSQA practice in Ontario and may not represent the practice in other regions of Canada. However, Ontario is the most populous province in Canada with over 40,000 cases treated per year. Furthermore, it has more than a quarter of all major cancer centers in Canada and has more than 100 linacs.

There may be, however, biases introduced by limiting the distribution of our survey to Ontario cancer centers only. First, there may be a sampling bias because the distribution of equipment from major vendors found in Ontario may not reflect that of all cancer centers. Second, most Ontario cancer centers benefit from the presence of physics assistants or associates who actually conduct the PSQA measurements as opposed to staff physicists. Finally, the Canadian context favors larger cancer centers, typically involving more than four linacs, and therefore helps streamline the PSQA workflow, policies, and procedures.

### 3.A Policy and procedure

When making recommendations about PSQA practice, it is important to consider the impact that performing these measurements has on utilization of IMRT and VMAT. When asked if PSQA activities prevented expansion of their IMRT and VMAT utilization (Supporting Information, A1), a simple majority of clinics stated that IMRT and VMAT was used for all sites that show a benefit, whereas five (33%) responded that the time required to develop IMRT and VMAT had delayed the further implementation of these techniques. From these responses PSQA was not likely a significant deterrent to increasing IMRT and VMAT utilization. However, a few respondents commented that the PSQA workload was a significant burden affecting physics, dosimetry, or treatment.

**Fig. 1:**
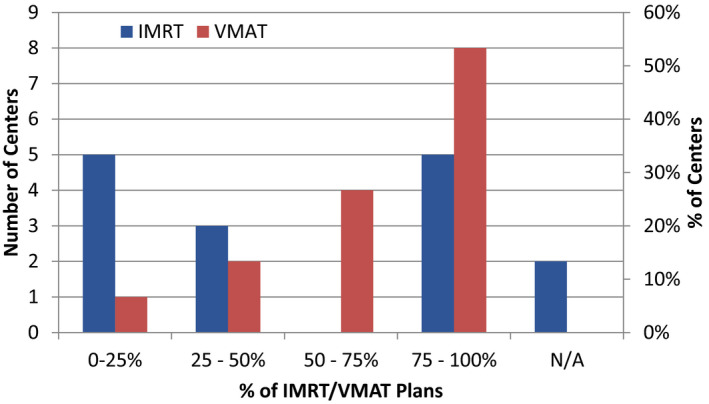
Distribution of center responses on percentage of IMRT and VMAT plans that utilized standard planning protocols such as same beam geometry, objectives, and planning avoidance based on developed or commissioned class solutions. The choice, “N/A”, refers to centers that do not treat with IMRT.

Standardized plan protocols or class solutions that utilize a consistent set of beam geometries and optimization objectives help improve efficiency and consistency of treatment plans and PSQA processes and results.^8^, ^9^, ^15^ Figure 1 shows the distribution of how the centers responded when asked about the percentage of IMRT and VMAT plans that utilized standardized planning protocols such as same beam geometry, objectives, and planning avoidance based on developed or commissioned class solutions (Supporting Information, A2, A3). Note that unlike IMRT, a large number of centers reported that they used standard approaches for VMAT planning for most of their plans. This may be related to the decrease in the utilization of IMRT relative to VMAT, as five centers responded that they rarely or never treat with IMRT.

**Fig. 2:**
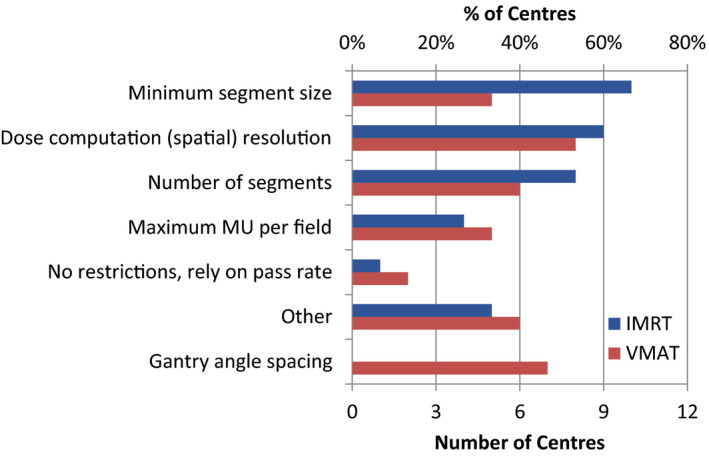
Distribution of center responses on which plan features were limited for IMRT and VMAT based on the impact they have on patient‐specific quality assurance (PSQA) results.

When developing standard plan protocols or class solutions, it is common practice to limit certain features within the planning system that might increase the plan complexity, which could result in low PSQA pass rates.^16^, ^17^ Figure 2 shows the frequency of plan features that the participating centers indicated were limited for IMRT and VMAT based on the impact that these have on the PSQA pass rates (Supporting Information, A4, A5). The most common limiting features reported were minimum segment size for IMRT and dose computation (spatial) resolution for VMAT. Two centers indicated in the comment that they also limited MU per cGy. There were very few centers (≤2) that relied solely on the pass rate of the PSQA measurement.

An important quality assurance task is to verify the correct transfer of data between the TPS, record, and verify system and the linac.^3^ When asked about methods used to verify the data transfer (Supporting Information, A6, A7), all centers indicated that they either had an integrated system (e.g., Aria, Varian Oncology Systems, CA) where no explicit data transfer took place or had procedures in place to verify correct data transfer. Of the 11 centers (73%) that performed PSQA measurements to verify the correct transfer of data, all but one also relied on at least one other check. Reported methods of data transfer check included manual checking of the basic beam parameters, automated scripts, and delivery logs. All centers but one indicated that procedures were in place to ensure that no accidental changes were made to the fields once correct transfer to the record and verify system was ensured (Supporting Information, A8). The remaining center indicated a gap in procedures was identified as a result of the survey, and the deficiency was being addressed.

**Fig. 3:**
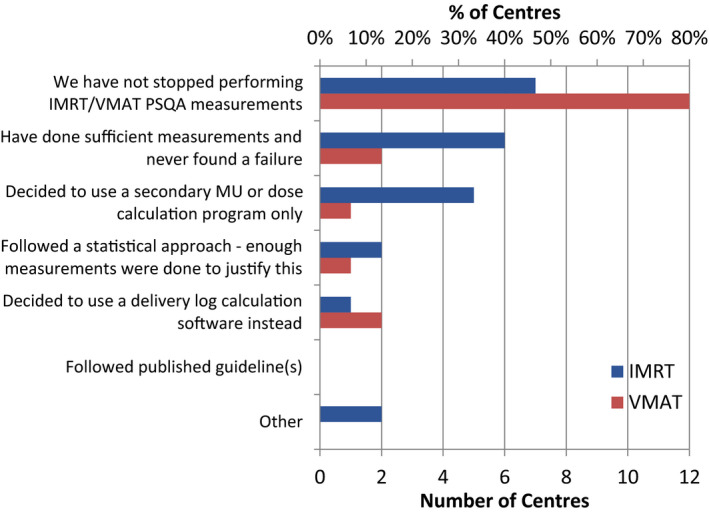
Decision‐making methodology to stop performing patient‐specific quality assurance (PSQA) measurements for a specific site, class solution, or technique.

When centers were asked about the methodology used to determine which plans required PSQA measurement or delivery log (Supporting Information, A9), 11 (73%) centers responded that they performed PSQA on all IMRT (if utilized) and VMAT plans. Some clinics reported that PSQA measurements were no longer acquired for specific sites, class solutions, or techniques (Supporting Information, A10, A11); Fig. 3 shows the justification for these decisions. Stopping PSQA practice was justified, for a minority of clinics, on the basis that sufficient measurements were done without any failures. However, it was left up to the individual respondents to determine how many measurements were necessary to support this decision. We note that few centers employed a data‐driven statistical approach to justify the decision to stop. The survey results indicate a need to have standardized recommendations providing systematic approaches for stopping or reducing frequency of PSQA measurements for mature techniques. Currently, there are very few recommendations on modifying the PSQA measurement frequency, ranging from the discretion of a qualified medical physicist with justification by a rigorous statistical analysis of existing data and documentation^10^ to the dependence on the level of institutional experience with IMRT and whether the class solution is existing or developing.^8^

### 3.B Measurement vs delivery log

Although PSQA is typically a measurement‐based process, there has been growing interest in substituting measurements with analysis of delivery log files.^18^, ^19^, ^20^ Delivery log analysis is advantageous for its potential to improve efficiency and reduce the workload associated with performing PSQA measurements. One significant difference between delivery log and measurement‐based methods is that the former relies on the self‐reported delivery parameters from the linac, rather than providing an independent assessment. There are a variety of techniques and commercial solutions available for both measurement‐based PSQA and delivery log analysis. The implementation of these techniques and instrumentation used can impact PSQA results.

**Fig. 4:**
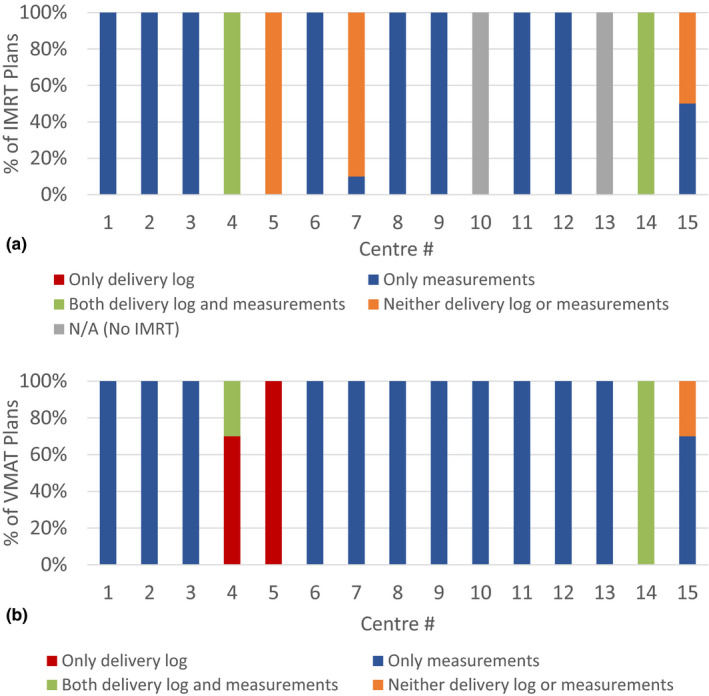
Percentage of (a) IMRT and (b) VMAT plans undergoing patient‐specific quality assurance using delivery log calculation software vs measurements.

Participants were surveyed on their use of delivery log analysis vs measurement‐based PSQA for both IMRT and VMAT treatment plans (Supporting Information, B1, B2, B3). As shown in Fig. 4a and b, the sole use of delivery log calculation software for PSQA was not widely implemented (one center for VMAT and none for IMRT). Of the 13 centers that delivered IMRT, only one performed no measurements for any IMRT plans. However, two indicated that they used both delivery logs and measurements for all IMRT plans. Similarly, only one center did not perform PSQA measurements for any VMAT plans, but used delivery log analysis instead. Nevertheless, eight (53%) respondents expressed interest in adopting delivery log analysis. This suggests that log file analysis may play a more important role in the PSQA process in the near future as centers transit to this approach.

### 3.C Instrumentation

Table 1 shows the distribution of devices used for PSQA measurements (Supporting Information, C1). Most commonly for both IMRT and VMAT measurements, pseudo‐3D detector arrays were used rather than flat 2D detectors. The Sun Nuclear ArcCHECK was the most popular, by a wide margin. In addition, three and four centers primarily used EPID dosimetry for IMRT and VMAT, respectively. However, we did not inquire which EPID dosimetry software or calculation algorithm, if applicable, was used to determine the measured dose. Four of the centers that used the ArcCHECK for VMAT also used other devices for some measurements (two used EPID, one used MapCHECK, and one used Delta4). This is in contrast to other surveys published, which reported that planar and point dose measurement outnumbered 3D detector arrays.^12^, ^13^

**Table 1:** Measurement devices used for IMRT and VMAT patient‐specific quality assurance (PSQA) measurements.

Measurement device	Number of centers (%)	
	IMRT	VMAT
SNC ArcCheck	8 (53%)	11 (73%)
ScandiDos Delta^4^	0 (0%)	2 (13%)
IBA MatriXX	2 (13%)	1 (7%)
SNC MapCheck/MapCheck2	2 (13%)	1 (7%)
EPID dosimetry	3 (20%)	4 (27%)
Film	0 (0%)	0 (0%)
Multiple devices	3 (20%)	4 (27%)

Note:

Two of the 15 surveyed centers did not treat with IMRT.

Before a detector is used for PSQA measurement, a calibration is often required to convert the measured reading to dose in a typically nonreference geometry. When asked how the calibration was performed (Supporting Information, C2), nine centers (60%) converted reading to dose calculated in the TPS based on a certain beam geometry, whereas three (20%) followed a standard dosimetric protocol for this calibration.

Regular quality control (QC) of the PSQA detector and software, including reproducibility and stability checks of simple fields or base plans, ensures that the devices perform optimally and accurately. The majority (nine centers or 60%) of the centers reported having regular QC (Supporting Information, C3). For the other centers, two commented performing ad hoc QC or as needed, whereas one stopped their regular QC because they never discovered any problems.

### 3.D Measurement setup and methodology

Results of PSQA have been shown to be sensitive to differences in measurement setup and methodology. For example, phantom setup for PSQA can be achieved with various geometries including TC, perpendicular field‐by‐field (PFF), and perpendicular composite (PC). The definitions of these setup geometries can be found in Task Group 218.^7^ Consideration should also be given to the choice of linac used for the PSQA measurement vs patient treatment, as well as accommodation for linac output variation. In addition, systematic dosimetric errors could be caused by phantom heterogeneities due to the presence of high atomic number components in the detectors and electronics.

With regard to phantom setup, respondents showed a strong tendency towards TC (Supporting Information, D1, D2). Almost all (14 centers or 93%) reported using TC as their preferred setup for VMAT, whereas 10 out of 13 centers that treated with IMRT used TC. Five and seven used PFF for VMAT and IMRT, respectively, and no site used PC. Several centers used a combination of TC and PFF depending on the available equipment and delivery details. This is consistent with recommendations made in the literature where it has been suggested that TC provides a more direct measurement of dose summation that most closely mimics patient treatment delivery,^7^ and that if TC is not suitable for the detector (e.g., EPID for portal dosimetry), the PFF method may be used. It should be noted that all centers that used portal dosimetry for PSQA reported using PFF, whereas one center used TC for all coplanar beams and PFF for noncoplanar ones.

When asked if PSQA was performed on the same linac as the unit intended for treatment (Supporting Information, D3), 12 (80%) reported the use of any beam‐matched linac. The remaining centers tried to perform measurement on the treatment linac unless it was not immediately available. Practicality and efficiency were cited as the rationale for not requiring measurement be carried out on the machine being used for the patient's treatment. The findings are consistent with the CPQR guidelines,^10^ and it has been shown that patient‐specific measurements can be done on any beam‐matched linac.^21^, ^22^ Consistent intermachine performance may be achieved by compliance to appropriate regular linac QC guidelines^23^, ^24^, ^25^ as well as periodic performance of a set of appropriate IMRT/VMAT specific tests^26^, ^27^ and/or by statistical process control.^28^ Although this survey did not address intermachine consistency QC, the results suggest that all centers were performing PSQA on an appropriate linac. In a future survey, it might be beneficial to include a question that probes for measurement setup consistency and compliance. For instance, is the setup of the device and alignment with linac isocenter verified by cone beam CT imaging?

Centers were asked whether they corrected their PSQA measurement dose to account for linac output variation (Supporting Information, D4). Excluding the three centers that used EPID dosimetry, 10 of 12 respondents reported correcting PSQA measurement for output variation by using output measured either immediately before or after the measurements (9/10) or during morning daily output QC (1/10). The other two respondents cited a desire to have a PSQA result that more accurately represented the dose that would be delivered to the actual patient. However, centers that take output variation into account may be less vulnerable to intermachine inconsistency.

On the handling of heterogeneity settings in the TPS, consideration should be made to account for CT artefacts caused by high atomic number detectors/electronics and incorrect densities interpretation for high atomic number detectors/electronics/phantom.^29^ When asked how they handle inhomogeneity caused by the detector on the CT scan (Supporting Information, D5), all centers using phantom‐based PSQA reported that they override the electron density within the planning system. Various methods of override were reported including using a vendor supplied virtual phantom or overriding the density in some or all of the CT phantom.

### 3.E Data analysis and interpretation

Two‐dimensional or 3D Gamma evaluation for dose difference and distance‐to‐agreement (DTA) in phantom are commonly used for verification of treatment plan delivery.^7^ In Gamma evaluation, the reference dose distribution is the one against which the evaluated dose distribution is compared, combining both the dose difference and the DTA between the two distributions into a single quantity. Composite analysis, a closely related concept to Gamma, evaluates the dose difference and the DTA separately between the two distributions. The chosen spatial resolution and dimensionality of both distributions can affect the reliability and accuracy of the PSQA analysis.^9^ Further, the choice of type of dose normalization (global or local, absolute or relative) as well as the point of normalization can greatly affect the resulting pass rates. In addition, vendor specific features such as auto grid shift, measurement uncertainty, and fast DTA algorithms to save computing time can affect passing rates in a way that can mask clinically significant problems. Regardless of methodology, the Gamma evaluation criteria and associated measurement devices should be chosen to be sensitive enough to detect clinically significant errors that can reveal deficiencies in treatment planning beam models and delivery. Setting evaluation criteria and pass rate tolerance and action levels for PSQA should be part of implementing a new IMRT or VMAT technique, class solution, or measurement procedure.

Participants were asked about their PSQA evaluation criteria for both IMRT and VMAT for common treatment sites (see Table 2) (Supporting Information, E1). For Gamma or composite analysis, irrespective of the sites, the most common responses were 3% dose difference, 3‐mm DTA, 10% low dose threshold (LDT), and 95% pass rate for both tolerance and action levels. Interestingly, they were also the most common responses from the national survey in China^12^ and the predominantly US–Canada survey conducted by the IROC‐Houston QA center.^13^ For comparison, Task Group 218 recommends 3%/2 mm, 10% LDT, and a universal 95% tolerance and 90% action levels,^7^ whereas the NCS code of practice recommends at least 3%/3 mm with the same LDT and action level.^8^, ^9^ When asked how these criteria were determined for Gamma/composite analysis (Supporting Information, E2) and pass tolerance/action levels (Supporting Information, E3), the results were almost evenly split, between the choices of, in‐house experience, published guidelines, and following medical physics community (53%, 47%, and 47%, respectively for Supporting Information, E2, and 40%, 33%, and 40%, respectively for Supporting Information, E3), suggesting no universal approach for IMRT or VMAT.

**Table 2:** Patient‐specific quality assurance (PSQA) evaluation and pass rate criteria for both IMRT and VMAT for four common treatment sites: head and neck, intact prostate, SBRT lung, and non‐SBRT palliative.

		% of centers			
		Head & neck	Intact prostate	SBRT lung	Palliative (non‐SBRT)
Dose difference	3%	73%	93%	87%	80%
	2%	0%	7%	7%	7%
Distance to agreement	3 mm	47%	67%	47%	60%
	2 mm	27%	33%	47%	33%
Low dose threshold	10%	60%	80%	73%	73%
	5%	7%	13%	13%	13%
Pass rate (tolerance)	95%	33%	53%	47%	53%
	90%	20%	20%	20%	20%
Pass rate (action)	95%	33%	40%	40%	33%
	90%	7%	20%	13%	20%

Abbreviation: SBRT, stereotactic body radiation therapy.

**Fig. 5:**
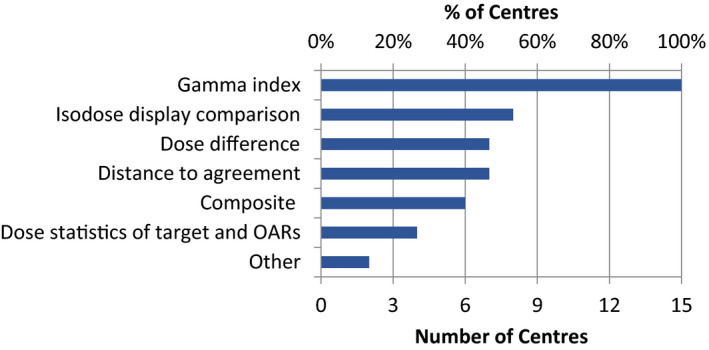
Methods of evaluation of patient‐specific quality assurance results.

**Fig. 6:**
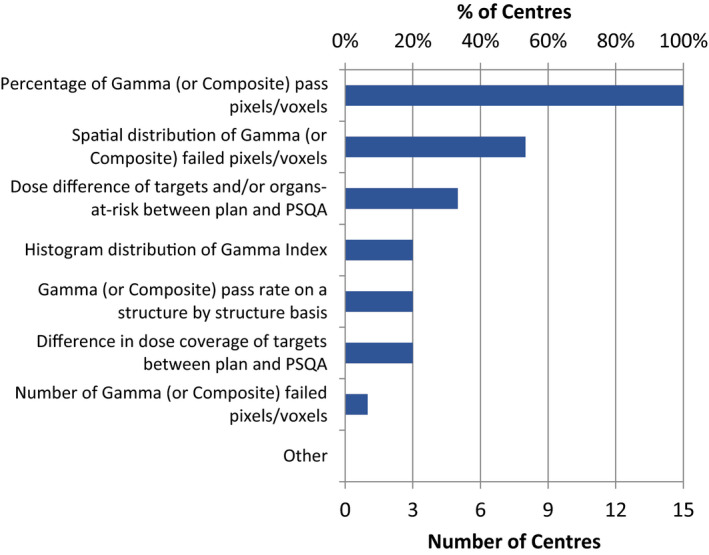
Determination of pass or fail of patient‐specific quality assurance results.

All centers considered Gamma and six (40%) centers also used composite analysis in evaluating PSQA results (Fig. 5) (Supporting Information, E4). As for PSQA pass determination (Fig. 6) (Supporting Information, E5), again all centers considered Gamma (or composite). Gamma analysis was also reported as the analysis method by the vast majority of respondents in other similar surveys.^12^, ^13^ In addition, eight (53%) centers also reviewed the spatial distribution of Gamma (or composite) failed pixels or voxels. However, very few centers examined the difference in dose distribution in targets or organs‐at‐risk, likely due to a lack of available software. In terms of dose normalization for dose difference or Gamma analysis, 11 (73%) used global, and four (27%) used local normalization in absolute dose (Supporting Information, E6).

It should be noted that the reported evaluation criteria should not be considered universal among all analysis software. As a case in point, 10 (67%) indicated they enabled a vendor specific option in their analysis on all measurements or at the discretion of the physicist or physics assistant (Supporting Information, E7). For example, six (40%) centers reported enabling “measurement uncertainty,” which effectively changes the dose difference criterion.

This wide variation in practice indicates the need for a more consistent analysis approach when comparing PSQA pass rates among centers.^3^ Perhaps to improve intraprovincial consistency, systematic guidelines^7^, ^11^ could be provided for each center to determine consistent analysis parameters and standardize tolerance and action levels for PSQA pass rates.

### 3.F Documentation, process, and feedback

The PSQA measurement is vulnerable to (a) variations in the execution of its associated process, (b) variations between users and how they interpret the result, and (c) the equipment itself. In order to produce reliable and reproducible results, the potential variations introduced at each step in this process have to be characterized and, as much as possible, minimized. Development of written procedures for measurement setup, delivery, and interpretation of results are crucial for the cohesiveness of the PSQA measurement process.^10^ Once the process is established, an independent validation by credentialing laboratories such as IROC can be sought to help gain insight into the quality of treatments compared with other centers what QA results are achievable.^3^, ^9^

For continuous quality improvement, periodic review and analysis of the pass rates can help identify the causes of low pass rates or failure.^7^ Methods from statistical process control, for example, can be employed to derive local tolerances and action levels and to detect outliers.^30^, ^31^, ^32^, ^33^ Furthermore, regular data review and analysis can help the physicist identify unwanted changes in the process and decide on the appropriate course of action.

The survey showed that the vast majority of centers had developed written procedures for PSQA measurement setup and delivery (100%), data preparation in the TPS (93%), measurement analysis (93%), and tolerance/action levels for pass rates (87%) (Supporting Information, F1). With the exception of one center that used delivery logs, PSQA tests were not performed or reviewed for every fraction (Supporting Information, F2). Among reasons cited were lack of resources or infrastructure, no evidence of value, or other QC tests that characterized machine performance. Two‐thirds of centers reviewed all or some PSQA results regularly (Supporting Information, F3). One center performed a control chart analysis for their recently implemented EPID dosimetry‐based PSQA process. Centers that did not review all PSQA results cited obstacles such as lack of automated extraction tools, lack of urgency due to historically high pass rates, and lack of benefits of regular and timely review.

**Fig. 7:**
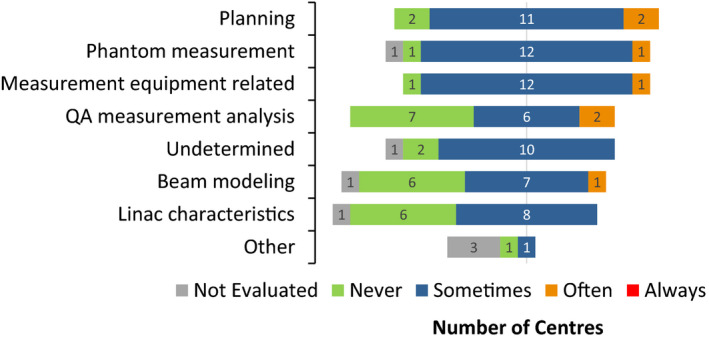
Frequency of causes of low pass rates or failures in patient‐specific quality assurance. “Planning” refers to various treatment plan settings or parameters, such as small segment size, number of small segments, inadequate optimization and large beam modulation. “Phantom measurement” has to do with such errors as incorrect phantom setup, shift, and plan transfer. “Measurement equipment” refers to problems such as changes in detector response over time and nonuniform response not properly corrected. “QA measurement analysis” has to do with problems such as using incorrect analysis parameters and poor registration of measured and calculated dose distribution. “Beam modeling” refers to poor modeling of the linac components, beam dose distribution and dosimetric parameters. Finally, “linac characteristics” refers to the various components of the machine that are out of tolerance, such as MLC position and speed, beam and couch angles, gantry speed, dose and dose rate linearity, and beam energy and symmetry.

Figure 7 illustrates the perceived frequency with which different factors caused PSQA failures or low pass rates in the different centers (Supporting Information, F4). Weighted average responses of the survey question were distributed approximately uniformly for all these listed factors, suggesting they were all relevant causes of PSQA failures or low pass rates. When asked about the most frequent source for PSQA failures and steps taken to improve it (Supporting Information, F5), six (40%) centers selected “Planning,” which refers to various treatment planning settings or parameters, as the most frequent factor for PSQA failures, followed by “Measurement equipment” (four centers or 27%), which refers to problems related to the equipment itself or its improper use. The finding is similar to that of the survey in China where the most frequent reason for failed PSQA is highly modulated plans.^12^ Three (20%) centers indicated they did not have any frequent factor for PSQA failures. Comments did not show a consistent pattern in actions taken to improve pass rates, suggesting that causes were center‐specific and varied with equipment, software and clinical practices. Among the comments, re‐planning (three) and few or no failures (three) were most cited. Answers were mainly confounded by the fact that there were usually very few PSQA failures.

**Fig. 8:**
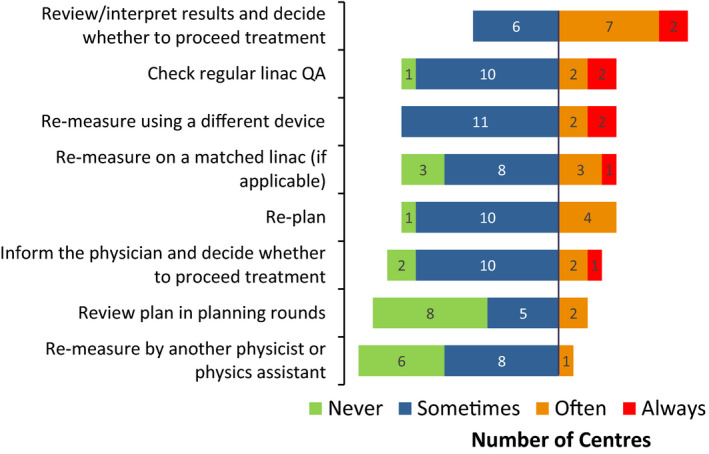
Course of action followed by the surveyed centers when a plan fails patient‐specific quality assurance.

In case of failed PSQA, all but one center discussed and learned from PSQA failures (Supporting Information, F6). The feedback mechanism varied widely among different centers, with many centers discussing PSQA failures informally. Four centers (27%) did not have a formalized course of action (Supporting Information, F1). Three centers commented that learning from PSQA failures had led to changes in planning practices and for one center, improvement of beam models. Figure 8 indicates that all courses of action taken when a plan failed PSQA were followed “Often” or “Sometimes” by at least one center (Supporting Information, F7). The weighted average of responses suggests that “Review/interpret results and decide whether to proceed to treatment” was the most frequent course of action, and “Review plan in planning rounds” was the least frequent. It also appears that there was some reluctance to replan or remeasure in many centers, as often there was perceived clinical pressure to get the treatment started in a timely manner. Similarly, the survey of centers in China^12^ and by the IROC‐Houston QA center^13^ both found that replanning ranked among the least popular options when responding to PSQA failures. Treatment delay may be avoided if a center specifies a timeline for completion of PSQA checks as well as actions to be taken when PSQA fails in its standard operating procedure.^3^

All sites kept some form of record of the PSQA results (Supporting Information, F8): nine (60%) kept records in a retrievable fashion, where data could be used for trending, analysis or comparison. Eleven (73%) kept an official record such as in a record and verify system. The results are in line with the guidelines from ACR/ASTRO^4^ and CPQR.^10^ All centers had participated in an independent credentialing process for IMRT and/or VMAT, such as IROC and/or the Ontario Health (Cancer Care Ontario) Collaborative Quality Assurance audit (Supporting Information, F9).

## 4 CONCLUSIONS

An extensive survey on PSQA practice for IMRT and VMAT delivery was completed by 15 Ontario cancer centers in Canada for the Ontario Health (Cancer Care Ontario) PCOP PSQA working group in early 2019. The results of this survey provided a snapshot of the current state of many aspects of PSQA‐related practice in Ontario and highlighted considerable provincial variations in PSQA practice. Among the major findings,

- All centers but one had procedures in place to ensure that no accidental changes were made to the fields once correct transfer to the record and verify system and/or linac was ensured. As a result of the survey, the deficiency in procedures was being addressed at the remaining center.
- The majority of centers surveyed performed PSQA measurements for all VMAT plans. Among centers that have stopped or reduced PSQA measurements, no clear decision‐making methodology was reported. This indicates a need for clear guidelines or recommendations for stopping or reducing frequency of PSQA measurements.
- The majority of centers surveyed employ pseudo‐3D array detectors with a TC geometry. This geometry is consistent with recommendations made in the literature that this approach provides a more direct measurement of dose summation mimicking patient treatment delivery.
- Although only 13% of centers surveyed employed delivery log evaluation at the time of the survey, 53% of respondents expressed interest in adopting this technology, indicating a more important PSQA role in the future, in particular, as an alternative for PSQA measurements.
- When performing Gamma (or composite) analysis, 3% dose difference, 3‐mm DTA, 10% LDT, and 95% pass rate were the most common responses for both tolerance and action levels. However, no universal approach was reported on how these criteria were determined. Further, many centers reported enabling vendor specific options during analysis that could lead to nonuniversal pass rates. This inconsistency in practice indicates the need for a systematic approach when comparing PSQA pass rates among centers.
- All but one center discussed and learned from PSQA failures. However, the feedback mechanism varied widely among different centers, with many centers discussing PSQA failures informally.
- Planning related issues such as using machine settings or beam parameters that could lead to an increase of complexity were cited to be the most frequent source for PSQA failures (40%). The most frequent course of action for a failed PSQA was to review the result and decide whether to proceed to treatment.

## CONFLICT OF INTEREST

No conflicts of interest.

## AUTHOR CONTRIBUTION

All authors have participated in the survey design, analysis of results, and/or the manuscript write up.

## Supporting information


**Data** **S1**. Summary of IMRT/VMAT PSQA survey results.
