# Supplementary material for: Survey of patient‐specific quality assurance practice for IMRT and VMAT
Source: J Appl Clin Med Phys. 2021 Jun 19;22(7):155–64. doi: 10.1002/acm2.13294 (PMC8292698; doi:10.1002/acm2.13294)
Supplement: Supplementary file 1 — Data S1. Summary of IMRT/VMAT PSQA survey results. [file ACM2-22-155-s001.pdf]

Supplement

A. Policy and Procedure

A1. Do PSQA activities prevent your centre from expanding the IMRT/VMAT practice?

Answer Choices	# of Centers	% of Centers
No, we already treat with IMRT/VMAT on all the sites that show a clinical benefit with these techniques	8	53%
No, but development of techniques and implementation takes time. So the adoption of IMRT/VMAT is gradual	5	33%
No, but longer IMRT/VMAT planning time reduces the number of patients we can treat without delay	1	7%
Yes, the high workload for PSQA activities makes it difficult to use more IMRT and VMAT in our practice	0	0%
Other	1	7%

A2. What percent (approximate) of IMRT plans (normalized to total number of IMRT plans) use standardized protocols (same beam geometry, objectives, planning avoidance, etc) based on developed or commissioned class solutions?

Answer Choices	# of Centers	% of Centers
0 - 25%	5	33%
25 - 50%	3	20%
50 - 75%	0	0%
75 - 100%	5	33%
N/A	2	13%

A3. What percent (approximate) of VMAT plans (normalized to total number of VMAT plans) use standardized protocols (same beam geometry, objectives, planning avoidance, etc) based on developed or commissioned class solutions?

Answer Choices	# of Centers	% of Centers
0 - 25%	1	7%
25 - 50%	2	13%
50 - 75%	4	27%
75 - 100%	8	53%
N/A	0	0%

A4. Have you defined restrictions or recommendations on the use of the following features in your planning system based on the impact these have on your IMRT PSQA pass rates (choose all that apply)?

Answer Choices	# of Centers	% of Centers
Minimum segment size	10	67%
Dose computation (spatial) resolution	9	60%
Number of segments	8	53%

Maximum MU per field	4	27%
No restrictions, rely on pass rate	1	7%
Other	5	33%

A5. Have you defined restrictions or recommendations on the use of the following features in your planning system based on the impact these have on your VMAT PSQA pass rates (choose all that apply)?

Answer Choices	# of Centers	% of Centers
Dose computation (spatial) resolution	8	53%
Gantry angle spacing	7	47%
Number of segments	6	40%
Maximum MU per field	5	33%
Minimum segment size	5	33%
No restrictions, rely on pass rate	2	13%
Other (please specify)	6	40%

A6. Do you have procedures to ensure consistency of the transfer of plan delivery parameters from the Treatment Planning System (TPS) to the Record & Verify (R & V) and/or linac?

Answer Choices	# of Centers	% of Centers
Yes, for all plans including 3D Conformal	12	80%
Yes, just for IMRT/VMAT plans	0	0%
No	3	20%

A7. How do you ensure the IMRT/VMAT beam transfer from the TPS to the R & V system or linac is correct (choose all that apply)?

Answer Choices	# of Centers	% of Centers
Indirect checking through PSQA measurement	11	73%
Manual checking between the TPS and R & V	10	67%
Automatic checking via a script between the TPS and R & V	4	27%
Indirect checking through delivery log	3	20%
Indirect checking through subjective comparison of measured fluence map	3	20%
Using vendor supplied treatment plan data integrity check (e.g. Varian's SCIC)	3	20%
Automatic checking via a script between the TPS and delivery log	0	0%
Other	2	13%

A8. After ensuring beam transfer from the TPS to R&V and/or linac is correct, what are your steps or procedures to ensure no alterations are accidentally made to the plan delivery parameters prior to treatment (choose all that apply)?

Answer Choices	# of Centers	% of Centers

Beam parameters are "locked" or "approved" in R&V	14	93%
Beam parameters are re-checked by therapists on treatment units	13	87%
PSQA measurement	9	60%
Indirect checking through subjective comparison of measured fluence map	4	27%
Verify beam parameters in delivery log during a "dry run"	2	13%
We do not have any procedures	0	0%
Other	1	7%

A9. How does your centre determine which IMRT or VMAT plan needs to get PSQA (choose all that apply)?

Answer Choices	# of Centers	% of Centers
We do PSQA on all IMRT and VMAT plans	11	73%
Depends on class solutions or delivery techniques	5	33%
Depends on disease sites	4	27%
Depends if the plan passes certain evaluation such as an MU calc or pre-delivery Gamma check	3	20%
Depends if certain plan parameters (e.g. MU/cGy) exceed their tolerances	3	20%
Depends on the delivery machine	1	7%
Other	1	7%

A10. If your centre has generally stopped doing IMRT PSQA measurements for a specific site, class solution, or technique, how was this decision made (choose all that apply)?

Answer Choices	# of Centers	% of Centers
We have not stopped performing IMRT PSQA measurements	7	47%
Have done sufficient measurements and never found a failure	6	40%
Decided to use a secondary MU or dose calculation program only	5	33%
Followed a statistical approach and enough measurements were done to justify this approach	2	13%
Decided to use a delivery log calculation software instead	1	7%
Followed published guideline(s)	0	0%
Other	2	13%

A11. If your centre has generally stopped doing VMAT PSQA measurements for a specific site, class solution, or technique, how was this decision made (choose all that apply)?

Answer Choices	# of Centers	% of Centers
We have not stopped performing VMAT PSQA measurements	12	80%
Have done sufficient measurements and never found a failure	2	13%
Decided to use a secondary MU or dose calculation program only	1	7%
Followed a statistical approach and enough measurements were done to justify this approach	1	7%

Decided to use a delivery log calculation software instead	2	13%
Followed published guideline(s)	0	0%
Other	0	0%

B. Measurement vs Delivery Log

B1. Do you use a delivery log calculation software for routine IMRT and VMAT PSQA?

Answer Choices	# of Centers	% of Centers
No, but we would like to implement delivery log calculation, but lacking software	7	47%
No interest in implementing delivery log calculation at this time	4	27%
Yes, for both IMRT and VMAT	2	13%
Yes, for VMAT only	1	7%
Yes, for IMRT only	0	0%
Other (No, but in the progress of commissioning delivery log software)	1	7%

B2. Indicate what percent (approximate) of your IMRT plans undergoing PSQA uses the following. Note: the percentages should add up to 100%.

	Percentage of IMRT plans		
Answer Choices | # of centres	0%	Between 0 and 100%	100%
Only delivery log calculation software	15	0	0
Only measurements	5	2	8
Both delivery and measurements	13	0	2
Neither delivery log or measurements	12	2	1
N/A (No IMRT)	13	0	2

B3. Indicate what percent (approximate) of your VMAT plans undergoing PSQA uses the following. Note: the percentages should add up to 100%.

	Percentage of IMRT plans		
Answer Choices | # of centres	0%	Between 0 and 100%	100%
Only delivery log calculation software	13	1	1
Only measurements	3	1	11
Both delivery and measurements	13	1	1
Neither delivery log or measurements	14	1	0

C. Instrumentation

C1. Which measurement device(s) and its measuring and analyzing software, if applicable, do you use for routine IMRT and VMAT PSQA?

Devices | # of Centers (%)	IMRT		VMAT		SBRT	
ArcCHECK	8	53%	11	73%	12	80%
EPID Dosimetry	3	20%	4	27%	2	13%
Ion Chamber	0	0%	0	0%	4	27%
DELTA[4]	0	0%	2	13%	2	13%
MapCHECK/MapCHECK2	2	13%	1	7%	2	13%
MatriXX	2	13%	1	7%	0	0%
Film	0	0%	0	0%	1	7%
N/A	2	13%	0	0%	0	0%
Multiple Devices	3	20%	4	27%	6	40%

C2. How is the detector calibration done (converting reading or charge into dose) for your PSQA device?

Answer Choices	# of Centers	% of Centers
Convert measured reading or charge to dose calculated in the TPS based on certain beam geometry	9	60%
Follow a protocol such as TG-51 to obtain an absolute dose	3	20%
Other	3	20%

C3. Do you perform regular QA on your PSQA device or software?

Answer Choices	# of Centers	% of Centers
Yes	9	60%
No	6	40%

C4. Do you use the same measurement device(s) and its measuring and analyzing software, if applicable, during the development and testing of a new IMRT/VMAT class solution as those in the routine PSQA after the same new class solution is clinically implemented?

Answer Choices	# of Centers	% of Centers
Yes, we use the same device/software before and after clinical implementation for every class solution	7	47%
No, we use more accurate and/or higher resolution detector(s) during the development	4	27%
The answer depends on the site, technique, or class solution	3	20%
Other	1	7%

D. Measurement Setup and Methodology

D1. How do you set up your phantom for IMRT measurements (if different setups are used, choose all that apply)?

Answer Choices	# of Centers	% of Centers
True Composite	10	67%
Perpendicular field-by-field	7	47%
Perpendicular Composite	0	0%
Other	2	13%

D2. How do you set up your phantom for VMAT measurements (if different setups are used, choose all that apply)?

Answer Choices	# of Centers	% of Centers
True Composite	14	93%
Perpendicular field-by-field	5	33%
Perpendicular Composite	0	0%
Other	1	7%

D3. If you have matched linacs, do you always perform PSQA measurements on the same linac as the treatment linac and what is the rationale for your choice?

Answer Choices	# of Centers	% of Centers
No, we do measurements on any matched linacs	12	80%
We try to perform measurements on the treatment linac unless it is not immediately available	3	20%
Yes, we always perform measurements on the treatment linac	0	0%
N/A since we do not have multiple matched linacs	0	0%

D4. Do you perform a dose calibration measurement compared against a standard dose to factor the variation of the detector response and linac output into the PSQA measurements?

Answer Choices	# of Centers	% of Centers
Yes, we measure output immediately before or after measurements	9	60%
No	5	33%
Yes, we use daily output in the morning	1	7%

D5. How do you handle inhomogeneity in the detector on the CT scan for your PSQA plan calculations? For example, air in a chamber or electronics for the detectors.

Answer Choices	# of Centers	% of Centers
Override the density in the heterogeneous regions to the surrounding density	12	80%
Let the TPS do its heterogeneity correction	1	7%

Not applicable, our detector and phantom are homogeneous	0	0%
Other	3	20%

E. Data Analysis and Interpretation

E1. What are your typical criteria in analyzing and evaluating PSQA results using Gamma or composite analysis? Enter typical values, or N/A if not applicable.

		# of Centres (%)			
		Head & Neck	Intact Prostate	SBRT Lung	Palliative (non-SBRT)
Dose difference	2%	0 (0%)	1 (7%)	1 (7%)	1 (7%)
	3%	11 (73%)	14 (93%)	13 (87%)	12 (80%)
	5%	0 (0%)	0 (0%)	0 (0%)	1 (7%)
Distance to agreement	2 mm	4 (27%)	5 (33%)	7 (47%)	5 (33%)
	3 mm	7 (47%)	10 (67%)	7 (47%)	9 (60%)
Low dose threshold	0%	1 (7%)	1 (7%)	1 (7%)	1 (7%)
	5%	1 (7%)	2 (13%)	2 (13%)	2 (13%)
	10%	9 (60%)	12 (80%)	11 (73%)	11 (73%)
Not measured/treated		4 (27%)	0 (0%)	1 (7%)	1 (7%)
Pass rate - tolerance level	80%	1 (7%)	0 (0%)	1 (7%)	1 (7%)
	88%	0 (0%)	1 (7%)	0 (0%)	0 (0%)
	90%	3 (20%)	3 (20%)	3 (20%)	3 (20%)
	95%	5 (33%)	8 (53%)	7 (47%)	8 (53%)
	97%	1 (7%)	2 (13%)	2 (13%)	1 (7%)
	N/A	5 (33%)	1 (7%)	2 (13%)	2 (13%)
Pass rate - action level	80%	1 (7%)	1 (7%)	1 (7%)	1 (7%)
	90%	1 (7%)	3 (20%)	2 (13%)	3 (20%)
	95%	5 (33%)	6 (40%)	6 (40%)	5 (33%)
	Other*	1 (7%)	1 (7%)	1 (7%)	1 (7%)
	N/A	7 (47%)	4 (27%)	5 (33%)	5 (33%)

* physicist's discretion

E2. In Gamma or composite analysis, how does your centre determine the evaluation criteria (dose difference/DTA/dose threshold)?

Answer Choices	# of Centers	% of Centers
In house experience	8	53%
Based on published guideline(s)	7	47%

Follow other centres in medical physics community for ease of comparison	7	47%
Other	1	7%

E3. In Gamma or composite analysis, how does your centre determine the pass tolerance and action levels?

Answer Choices	# of Centers	% of Centers
In house experience	6	40%
Follow other centres in medical physics community for ease of comparison	6	40%
Universal tolerance based on published guideline(s)	5	33%
Based on statistical process control	4	27%
Other	0	0%

E4. How do you evaluate PSQA results (choose all that apply)?

Answer Choices	# of Centers	% of Centers
Gamma index	15	100%
Isodose display comparison	8	53%
Dose difference	7	47%
Distance to agreement	7	47%
Composite	6	40%
DVH, min, max, and mean dose of target and OARs	4	27%
Other	2	13%

E5. How do you determine if a PSQA passes (choose all that apply)?

Answer Choices	# of Centers	% of Centers
Percentage of Gamma (or Composite) pass pixels/voxels	15	100%
Spatial distribution of Gamma (or Composite) failed pixels/voxels	8	53%
Dose difference (e.g. mean or median) of targets and/or organs-at-risk between plan and PSQA	5	33%
Difference in dose coverage of targets (e.g. D95%) between plan and PSQA	3	20%
Gamma (or Composite) pass rate on a structure by structure basis	3	20%
Histogram distribution of Gamma Index	3	20%
Number of Gamma (or Composite) failed pixels/voxels	1	7%
Other	0	0

E6. How do you perform normalization in dose difference (or Gamma) analysis?

Answer Choices	# of Centers	% of Centers
Global normalization in absolute dose	11	73%

Local normalization in absolute dose	4	27%
Global normalization in relative dose	0	0%
Local normalization in relative dose	0	0%
N/A	0	0%

E7. Do you apply optional vendor specific features (such as autoshift, measurement uncertainty) that can affect plan comparison results?

Answer Choices	# of Centers	% of Centers
Yes, we apply vendor specific features on all measurements	6	40%
No, we do not apply any vendor specific features	5	33%
Yes, but at the discretion of the physicist or physics assistant doing the evaluation	4	27%

F. Documentation, Process and Feedback

F1. Has your centre created documents detailing the procedures for the following (choose all that apply)?

Answer Choices	# of Centers	% of Centers
Phantom setup and delivery for PSQA measurements	15	100%
Software settings for analysis of PSQA measurements	14	93%
How to prepare the data in the TPS	14	93%
Tolerance/Action levels for pass rates	13	87%
Course of action with a failed PSQA	11	73%
How to perform calculations and analysis from machine delivery logs, if applicable	4	27%
None of the above	0	0%

F2. Do you do PSQA (delivery log calculations or measurements using detectors such as an EPID or a transmission detector) and review the results for every fraction?

Answer Choices	# of Centers	% of Centers
No	14	93%
Yes, the results are based on delivery logs	1	7%
Yes, the results are based on measurements	0	0%

F3. Does your centre review PSQA results across patients, especially for the same disease sites, or class solutions, regularly to look for systematic errors in the system?

Answer Choices	# of Centers	% of Centers
Yes, we review PSQA results across all patients regularly	5	33%
Yes, we only review some PSQA results	5	33%
No	5	33%

Other	0	0%

F4. The following factors have been shown to affect pass rates of PSQA. Please indicate how often these factors, if evaluated/investigated, have been found to be a reason for PSQA failures or low pass rates at your centre.

Answer Choices | # of Centers (%)	Always		Often		Sometimes		Never		Not Evaluated	
Planning	0	0%	2	13%	11	73%	2	13%	0	0%
Phantom measurement	0	0%	1	7%	12	80%	1	7%	1	7%
Measurement equipment related	0	0%	1	7%	12	80%	1	7%	0	0%
QA measurement analysis	0	0%	2	13%	6	40%	7	47%	0	0%
Undetermined	0	0%	0	0%	10	67%	2	13%	1	7%
Beam modeling	0	0%	1	7%	7	47%	6	40%	1	7%
Linac characteristics	0	0%	0	0%	8	53%	6	40%	1	7%
Other	0	0%	0	0%	1	7%	1	7%	3	20%

F5. Of the following 4 factors, a) beam modelling, b) planning, c) linac characteristics, and d) measurement equipment listed in the previous question, please indicate the most frequent factor for PSQA failures or low pass rates, and the steps taken to remove it. Alternatively, if no action is taken, please indicate reasoning.

Answer Choices	# of Centers	% of Centers
Planning	6	40%
Measurement equipment	4	27%
We do not have any frequent factor for PSQA failures	3	20%
Beam modelling	1	7%
Linac characteristics	1	7%

F6. Does your centre discuss and learn from PSQA failures?

Answer Choices	# of Centers	% of Centers
Yes	14	93%
No	1	7%

F7. To which degree does your centre follow the course of action below when a plan fails PSQA?

Answer Choices | # of Centers (%)	Always		Often		Sometimes		Never	
Re-measure by another physicist or physics assistant	0	0%	1	7%	8	53%	6	40%
Review plan in planning rounds	0	0%	2	13%	5	33%	8	53%
Inform the physician and decide whether to proceed treatment	1	7%	2	13%	10	67%	2	13%
Re-plan	0	0%	4	27%	10	67%	1	7%

Re-measure on a matched linac (if applicable)	1	7%	3	20%	8	53%	3	20%
Re-measure using a different device	2	13%	2	13%	11	73%	0	0%
Check regular linac QA	2	13%	2	13%	10	67%	1	7%
Review/interpret results and decide whether to proceed treatment	2	13%	7	47%	6	40%	0	0%

F8. Does your centre keep recent records of PSQA (measurements + delivery log calculations) results?

Answer Choices	# of Centers	% of Centers
A). Yes, in a database or spreadsheet in a way that is easily retrievable for trending, analysis or comparison	4	27%
B). Yes, in patient's official records (such as R & V system)	6	40%
A). & B).	5	33%
No	0	0%

F9. Has your centre participated in an independent credentialing process for IMRT and/or VMAT (IROC for example)?

Answer Choices	# of Centers	% of Centers
Yes	15	100%
No	0	0%

G. Miscellaneous

G1. We would like to know your opinion regarding your centre's current IMRT/VMAT PSQA program, whether it is effective (such as the ability to catch a serious error), informative (e.g. to gain enough information to improve the planning and delivery), and efficient (please choose all that apply).

Answer Choices	# of Centers	% of Centers
Effective	15	100%
Informative	13	87%
Efficient	7	47%
Not effective, informative, or efficient	0	0%
Other	3	20%
